# Publisher Correction: Oncogenic hijacking of a developmental transcription factor evokes vulnerability toward oxidative stress in Ewing sarcoma

**DOI:** 10.1038/s41467-020-20017-2

**Published:** 2020-11-23

**Authors:** Aruna Marchetto, Shunya Ohmura, Martin F. Orth, Maximilian M. L. Knott, Maria V. Colombo, Chiara Arrigoni, Victor Bardinet, David Saucier, Fabienne S. Wehweck, Jing Li, Stefanie Stein, Julia S. Gerke, Michaela C. Baldauf, Julian Musa, Marlene Dallmayer, Laura Romero-Pérez, Tilman L. B. Hölting, James F. Amatruda, Andrea Cossarizza, Anton G. Henssen, Thomas Kirchner, Matteo Moretti, Florencia Cidre-Aranaz, Giuseppina Sannino, Thomas G. P. Grünewald

**Affiliations:** 1 Max-Eder Research Group for Pediatric Sarcoma Biology, Institute of Pathology, Faculty of Medicine, LMU Munich, Munich, Germany; 2 Institute of Pathology, Faculty of Medicine, LMU Munich, Munich, Germany; 3 Regenerative Medicine Technologies Laboratory, Ente Ospedaliero Cantonale (EOC), Lugano, Switzerland; 4 Department of Pediatrics, Division of Oncology and Hematology, Charité Berlin, Berlin, Germany; 5 Department of Pediatrics and Molecular Biology, University of Texas Southwestern Medical Center and Children’s Medical Center, Dallas, TX USA; 6 Department of General, Visceral and Transplantation Surgery, University of Heidelberg, Heidelberg, Germany; 7 Hopp-Children’s Cancer Center (KiTZ), Heidelberg, Germany; 8 Division of Translational Pediatric Sarcoma Research, German Cancer Research Center (DKFZ), Heidelberg, Germany; 9 Children’s Hospital of Los Angeles, Los Angeles, CA USA; 10 Department of Medical and Surgical Sciences for Children and Adults, University of Modena and Reggio Emilia School of Medicine, Modena, Italy; 11 Berlin Institute of Health, Berlin, Germany; 12 Experimental and Clinical Research Center (ECRC) of the MDC and Charité Berlin, Berlin, Germany; 13 German Cancer Consortium (DKTK), partner site, Berlin, Germany; 14 German Cancer Research Center (DKFZ), Heidelberg, Germany; 15 German Cancer Consortium (DKTK), partner site, Munich, Germany; 16 Institute of Pathology, University Hospital Heidelberg, Heidelberg, Germany

**Keywords:** Bone cancer, Targeted therapies, Paediatric cancer, Oncogenes, Sarcoma

Correction to: *Nature Communications* 10.1038/s41467-020-16244-2, published online 15 May 2020.

The original version of this Article contained an error in Fig. 3b. In Fig. 3b the “<” symbol in the labelling for light grey circles was replaced by “>”. This has been corrected in both the PDF and HTML versions of the Article.

