# Supplementary material for: Oncogenic hijacking of a developmental transcription factor evokes vulnerability toward oxidative stress in Ewing sarcoma
Source: Nat Commun. 2020 May 15;11:2423. doi: 10.1038/s41467-020-16244-2 (PMC7228971; doi:10.1038/s41467-020-16244-2)
Supplement: Supplementary file 1 — Supplementary Information [file 41467_2020_16244_MOESM1_ESM.pdf]

Supplementary Information

Oncogenic hijacking of a developmental transcription factor evokes vulnerability toward oxidative stress in Ewing sarcoma

Marchetto *et al.*

Supplementary Figure 1 Marchetto *et al.*

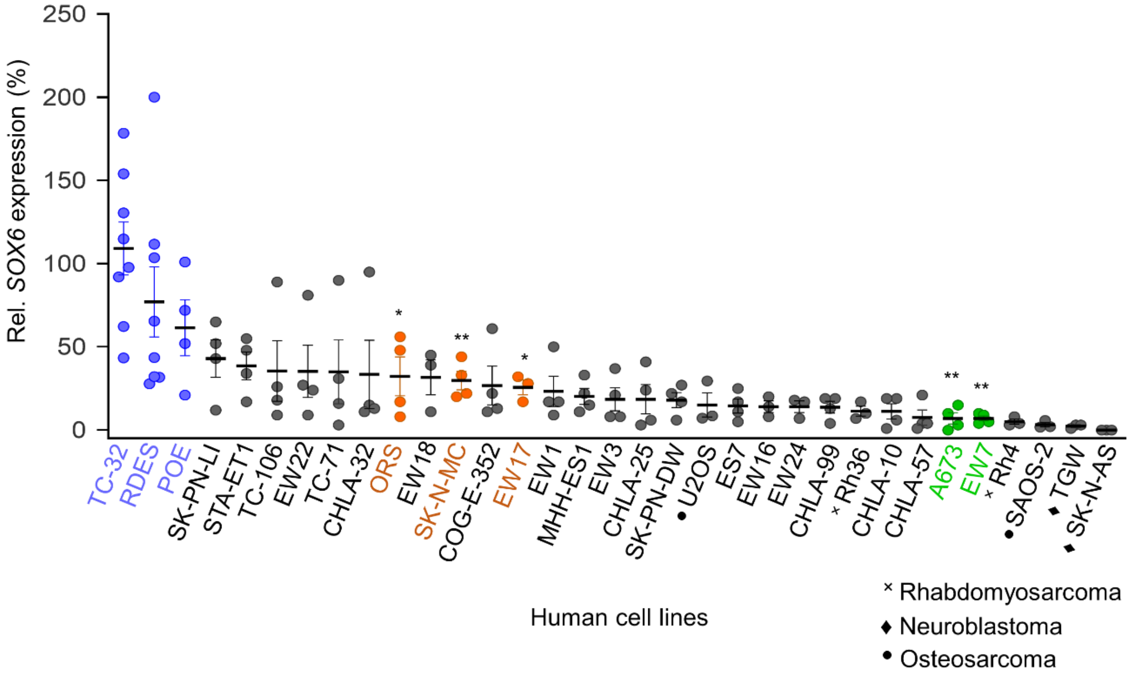

Supplementary Fig. 1 | Expression pattern of *SOX6* in pediatric cancer cell lines

SOX6 expression (qRT-PCR) of different EwS cell lines (*n* indicates the number of biologically independent experiments per cell line: TC-32 (*n*=8), RDES (*n*=8), POE (*n*=4), SK-PN-LI (*n*=4), STA-ET1 (*n*=4), TC-106 (*n*=4), EW22 (*n*=4), TC-71 (*n*=4), CHLA-32 (*n*=4), ORS (*n*=4, *P*=0.016), EW18 (*n*=3), SK-N-MC (*n*=4, *P*=0.008), COG-E-352 (*n*=4), EW17 (*n*=3, *P*=0.012), EW1 (*n*=4), MHH-ES1 (*n*=4), EW3 (*n*=4), CHLA-25 (*n*=4), SK-PN-DW (*n*=4), ES7 (*n*=4), EW16 (*n*=3), EW24 (*n*=3), CHLA-99 (*n*=4), CHLA-10 (*n*=4), CHLA-57 (*n*=4), A673 (*n*=4, *P*=0.004), EW7 (*n*=4, *P*=0.004)), rhabdomyosarcoma cell lines (*n* indicates the number of biologically independent experiments per cell line: Rh4 (*n*=3), Rh36 (*n*=3)), neuroblastoma cell lines (*n* indicates the number of biologically independent experiments per cell line: TGW (*n*=3), SK-N-AS (*n*=3)), and osteosarcoma cell lines (*n* indicates the number of biologically independent experiments per cell line: SAOS-2 (*n*=3), U2OS (*n*=3)). Expression levels were normalized to that of TC-32. Cell lines highlighted in blue color indicate EwS cell lines with highest *SOX6* expression that were used for the majority of experiments. Cell lines indicated in orange and green color represent EwS cell lines with intermediate or low *SOX6* expression, respectively. Horizontal bars represent means and whiskers SEM. *P* values determined via two-sided Mann-Whitney test by comparing TC-32 with the indicated cell line. **$P<0.01$, *$P<0.05$. Source data are provided as a Source Data file.

Supplementary Figure 2 Marchetto *et al.*

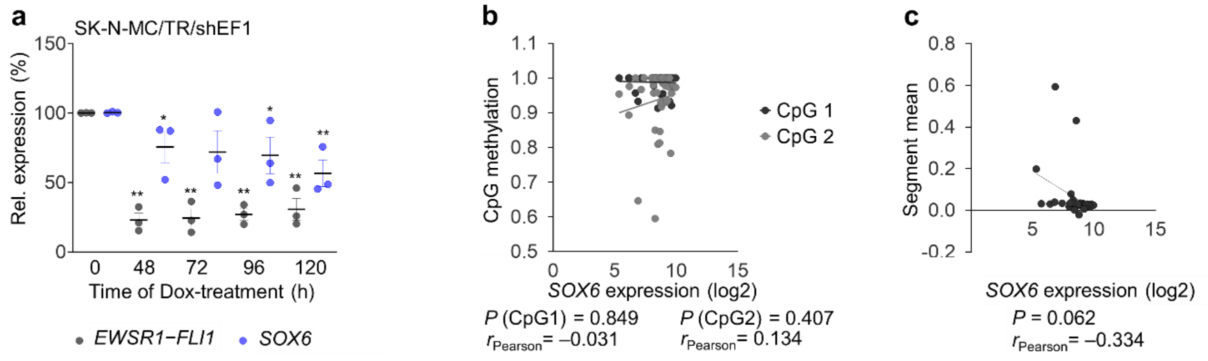

Supplementary Fig. 2 | *SOX6* is regulated by EWSR1-FLI1 in EwS but neither by differential promoter methylation nor copy number variations

a) *EWSR1-FLI1* and *SOX6* expression (qRT-PCR) in SK-N-MC/TR/shEF1 cells. Horizontal bars represent means and whiskers SEM, *n*=3 biologically independent experiments. *P* values determined via two-sided Mann-Whitney test by comparing the time point 0h with the indicated time points (EWSR1-FLI1: 48-120h *P*=0.002; SOX6: 48h *P*=0.015, 96h *P*=0.041, 120h *P*=0.002). ***P*<0.01, **P*<0.05. b) Correlation of methylation levels of two CpG-methylation sites within the *SOX6* promoter (CpG1/2) and *SOX6* expression levels (log2) in *n*=40 biologically independent primary EwS tumors. Lines indicate linear regressions of the data. *P* values determined via two-sided Pearson correlation test. c) Correlation of copy number variation (segment mean) at the *SOX6* locus with *SOX6* expression levels (log2) in *n*=32 biologically independent primary EwS tumors. The line indicates the linear regressions of the data. *P* values determined via two-sided Pearson correlation test. Source data are provided as a Source Data file.

Supplementary Figure 3 Marchetto *et al.*

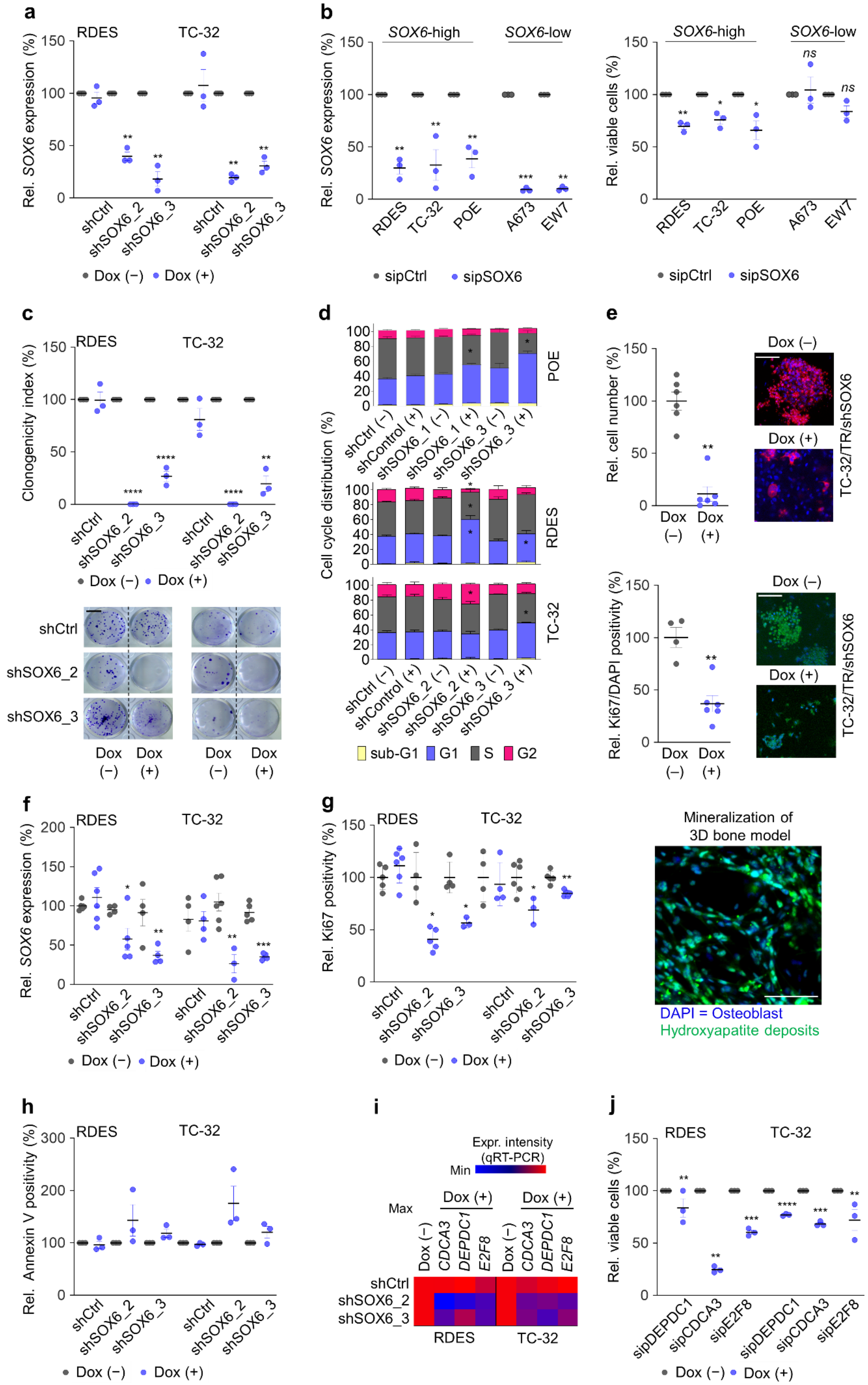

Supplementary Fig. 3 | *SOX6* promotes proliferation and growth of EwS cells

a) Relative *SOX6* expression (qRT-PCR) 96h after *SOX6* knockdown (shSOX6). Horizontal bars represent means, *n*=3 biologically independent experiments. *P* values determined via two-sided Mann-Whitney test (all *P*=0.002). b) Left: Relative *SOX6* expression in A673 (*P*=0.0002), EW7 (*P*=0.002), POE (*P*=0.002), RDES (*P*=0.002) and TC-32 (*P*=0.022) cells 96h after transfection with sipool-mediated *SOX6* knockdown. Horizontal bars represent means, *n*=3 biologically independent experiments. *P* values determined via two-sided Mann-Whitney test. Right: Viable cell count of the same cells. Horizontal bars represent means, *n*=3 biologically independent experiments, RDES (*P*=0.007), TC-32 (*P*=0.025), POE (*P*=0.047). *P* values determined via two-sided independent one-sample *t*-test. c) Upper: Clonogenicity index during 12d of *SOX6* knockdown. Horizontal bars represent means, *n*=3 biologically independent experiments, RDES: shSOX6_2 and shSOX6_3 (both *P*≤0.0001), TC-32: shSOX6_2 (*P*≤0.0001) and shSOX6_3 (*P*=0.009). *P* values determined via two-sided Mann-Whitney test. Lower: Representative micrographs; scale bar=1cm. d) Cell cycle phases after *SOX6* knockdown. Horizontal bars represent means (*n* indicates biologically independent experiments per group, RDES: shCtrl/shSOX6_2 (*n*=4), shSOX6_3 (*n*=3); TC-32: shCtrl/shSOX6_2 (*n*=4), shSOX6_3 (*n*=3); POE: (*n*=4). POE: S-phase shSOX6_1 (*P*=0.050), shSOX6_3 (*P*=0.029); RDES: G1 shSOX6_2 (*P*=0.0284), shSOX6_3 (*P*=0.0477), S-phase (*P*=0.029), G2 (*P*=0.027); TC-32: S-phase (*P*=0.050), G2 (*P*=0.027)). *P* values determined via two-sided Mann-Whitney test. e) Top: Relative number of TC-32/TR/shSOX6 cells. Horizontal bars represent means, *n*=6 biologically independent experiments, *P*=0.002. Representative micrographs of TC-32/TR/shSOX6 EwS cells (red) and osteoblasts (blue); scale bar=50µm. *P* values determined via two-sided Mann-Whitney test. Middle: Relative proliferation (Ki67/DAPI positivity) of TC-32/TR/shSOX6 cells in a 3D mineralized bone model. Horizontal bars represent means (*n* indicates biologically independent experiments per group, Dox(−) *n*=4, Dox(+) *n*=6, *P*=0.010). Representative micrographs of Ki67 staining of EwS cells (green) and osteoblasts (blue); scale bar=50µm. Bottom: Representative micrographs of mineralized bone matrix (blue=DAPI, green=hydroxyapatite); scale bar=100µM. f) *SOX6* expression (qRT-PCR) in RDES and TC-32 xenografts. Horizontal bars represent means (*n* indicates biologically independent xenografts, RDES: shCtrl (Dox(−) *n*=5, Dox(+) *n*=6), shSOX6_2 (Dox(−) *n*=4, Dox(+) *n*=5, *P*=0.027), shSOX6_3 *n*=4, *P*=0.002; TC-32: shCtrl *n*=4, shSOX6_2 (Dox(−) *n*=6, Dox(+) *n*=3, *P*=0.001), shSOX6_3 *n*=5, *P*≤0.0001). *P* values determined via two-sided Mann-Whitney test. g) Ki67 positivity of RDES and TC-32 xenografts. Horizontal bars represent means (*n* indicates biologically independent xenografts, RDES: shCtrl (Dox(−) *n*=5, Dox(+) *n*=6), shSOX6_2 (Dox(−) *n*=4, Dox(+) *n*=5, *P*=0.016), shSOX6_3 (Dox(−) *n*=4, Dox(+) *n*=3, *P*=0.050); TC-32: shCtrl *n*=4, shSOX6_2 (Dox(−) *n*=6, Dox(+) *n*=3, *P*=0.038), shSOX6_3 (*n*=5, *P*=0.008)). *P* values determined via two-sided Mann-Whitney test. h) Relative Annexin V positivity 96h after *SOX6* silencing. Horizontal bars represent means, *n*=3 biologically independent experiments. i) Relative *CDCA3*, *DEPDC1* and *E2F8* expression 96h after *SOX6* knockdown; averaged data of *n*=3 biologically independent experiments. j) Proliferation of EwS cells 96h after sipool-mediated knockdown of *CDCA3* (TC-32 *P*=0.0002, RDES *P*=0.002), *DEPDC1* (TC-32 *P*≤0.0001, RDES *P*=0.006) or *E2F8* (TC-32 *P*=0.010, RDES *P*=0.0006). Horizontal bars represent means, *n*=3 biologically independent experiments. *P* values determined via two-sided independent one-sample *t*-test. All error bars represent SEM. ****$P≤0.0001$, ***$P<0.001$, **$P<0.01$, *$P<0.05$. Source data are provided as a Source Data file.

Supplementary Figure 4 Marchetto *et al.*

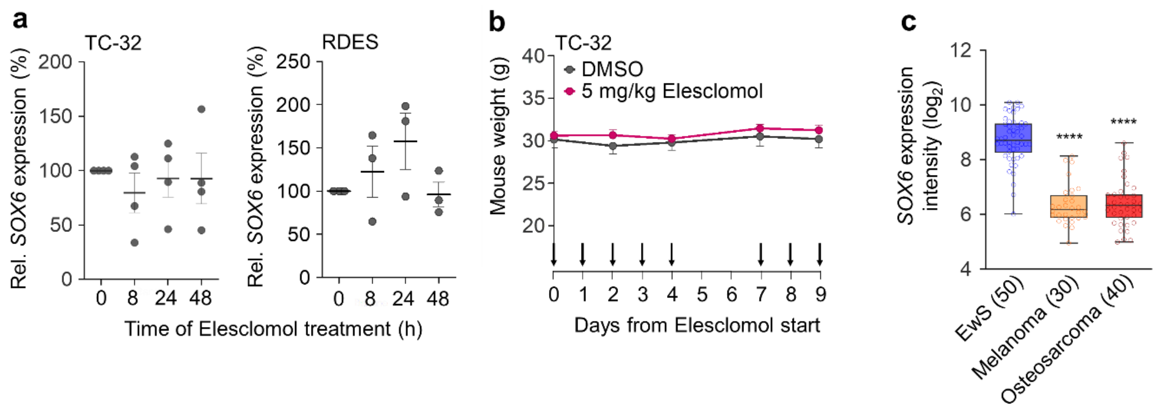

Supplementary Fig. 4 | Elesclomol has no effect on *SOX6* expression and mouse weight in *SOX6*-high EwS cells

a) Relative *SOX6* expression levels in RDES and TC-32 cells during Elesclomol-treatment (10 nM). Horizontal bars represent means and whiskers SEM, TC-32: *n*=4 biologically independent experiments, RDES: *n*=3 biologically independent experiments. b) Body weight of mice during intravenous Elesclomol-treatment (5 mg/kg). Dots represent means and whiskers SEM, *n*=5 biologically independent animals per condition. c) *SOX6* expression intensities (log2) of primary EwS tumors, melanomas ($P \leq 0.0001$) and osteosarcomas ($P \leq 0.0001$) as determined by Affymetrix microarrays[20]. The number of biologically independent samples (*n*) is given in parentheses. Horizontal bars indicate medians and boxes the interquartile range. Whiskers indicate the 10th and 90th percentile. *P* values determined via two-sided Mann-Whitney test. ****$P \leq 0.0001$. Source data are provided as a Source Data file.

Supplementary Figure 5 Marchetto *et al.*

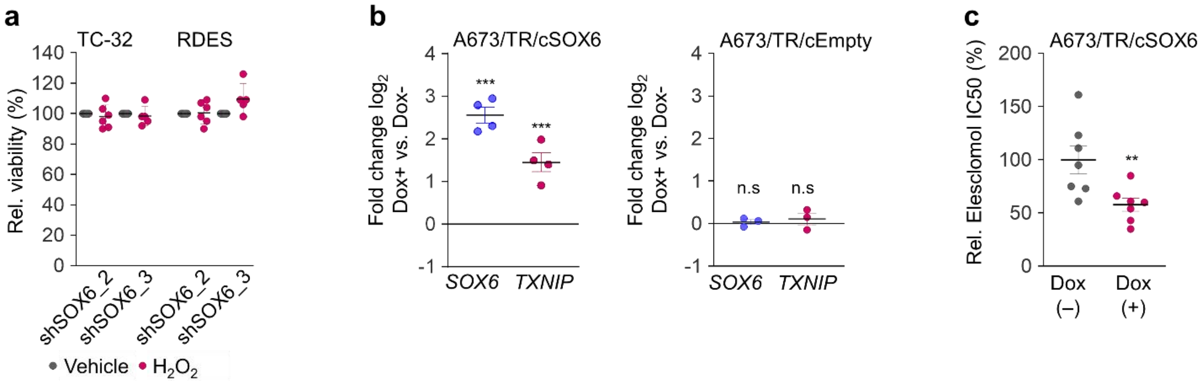

Supplementary Fig. 5 | *SOX6* overexpression induces *TXNIP* and Elesclomol sensitivity
a) Cell viability of EwS cells treated with vehicle (H_2O) or H_2O_2 (30 µM). Horizontal bars represent means (*n* indicates the number of biologically independent experiments, TC-32: shSOX6_2 *n*=6, shSOX6_3 *n*=5; RDES: shSOX6_2 *n*=6, shSOX6_3 *n*=5). b) Expression fold change (log2, qRT-PCR) of *SOX6* (*P*=0.0002) and *TXNIP* (*P*=0.0002) after 96h of *SOX6* overexpression in A673/TR/cSOX6 cells. Horizontal bars represent means, *n*=4 biologically independent experiments. *P* values determined via two-sided Mann-Whitney test. c) Relative Elesclomol IC50 in A673/TR/cSOX6 cells after 72h of concomitant Elesclomol and Dox-treatment. Horizontal bars represent means, *n*=7 biologically independent experiments, *P*=0.008. *P* values determined via two-sided Mann-Whitney test. All error bars represent SEM. ****P*<0.001, ***P*<0.01. Source data are provided as a Source Data file.

Supplementary Figure 6 Marchetto *et al.*

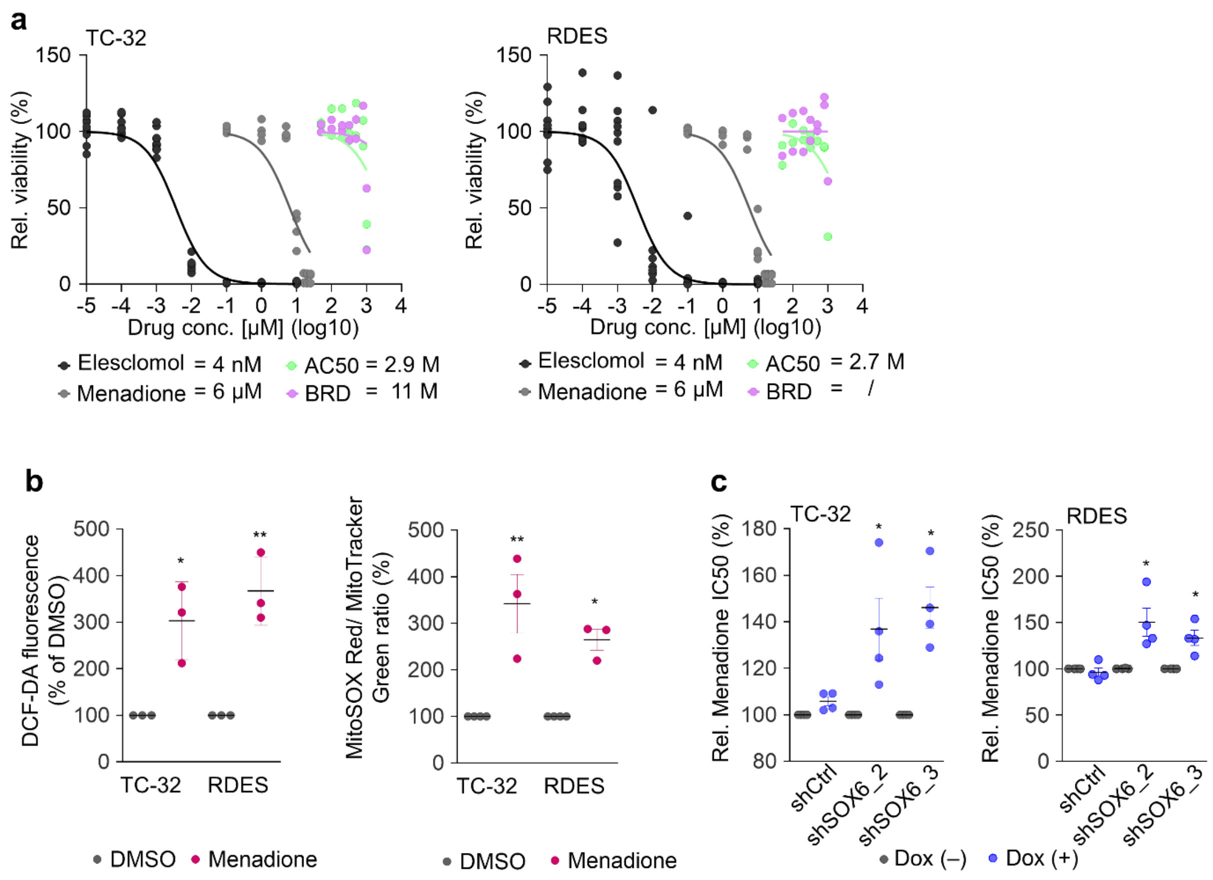

Supplementary Fig. 6 | Elesclomol is a potent inducer of oxidative stress in EwS

a) Relative viability of TC-32 and RDES cells after treatment with three different oxidative stress inducers (Menadione: TC-32 *n*=4, RDES *n*=4; DC_AC50 (AC50): TC-32 *n*=2, RDES *n*=2; BRD56491 (BRD): TC-32 *n*=2, RDES *n*=2) for 72h compared to Elesclomol (TC-32 *n*=9, RDES *n*=10), *n* indicates the number of biologically independent experiments per drug. Modeled dose-response curves and calculated IC50 values are displayed. b) Relative DCF-DA fluorescence (left) and MitoSOX Red/MitoTracker Green ratio (right) in TC-32 and RDES cells after treatment with Menadione (10 µM) compared to DMSO (control). Horizontal bars indicate means, *n*=3 biologically independent experiments. *P* values determined via two-sided independent one sample *t*-test (TC-32: DCF-DA *P*=0.047, MitoSOX Red *P*=0.014; RDES: DCF-DA *P*=0.004, MitoSOX Red *P*=0.004). c) Relative Menadione IC50 values in indicated cell lines 72h after start of concomitant Menadione- and Dox-treatment. Horizontal bars represent means, *n*=4 biologically independent experiments. *P* values determined via two-sided Mann-Whitney test (all *P*=0.029). All error bars represent SEM. **$P<0.01$, *$P<0.05$. Source data are provided as a Source Data file.

Supplementary Figure 7 Marchetto *et al.*

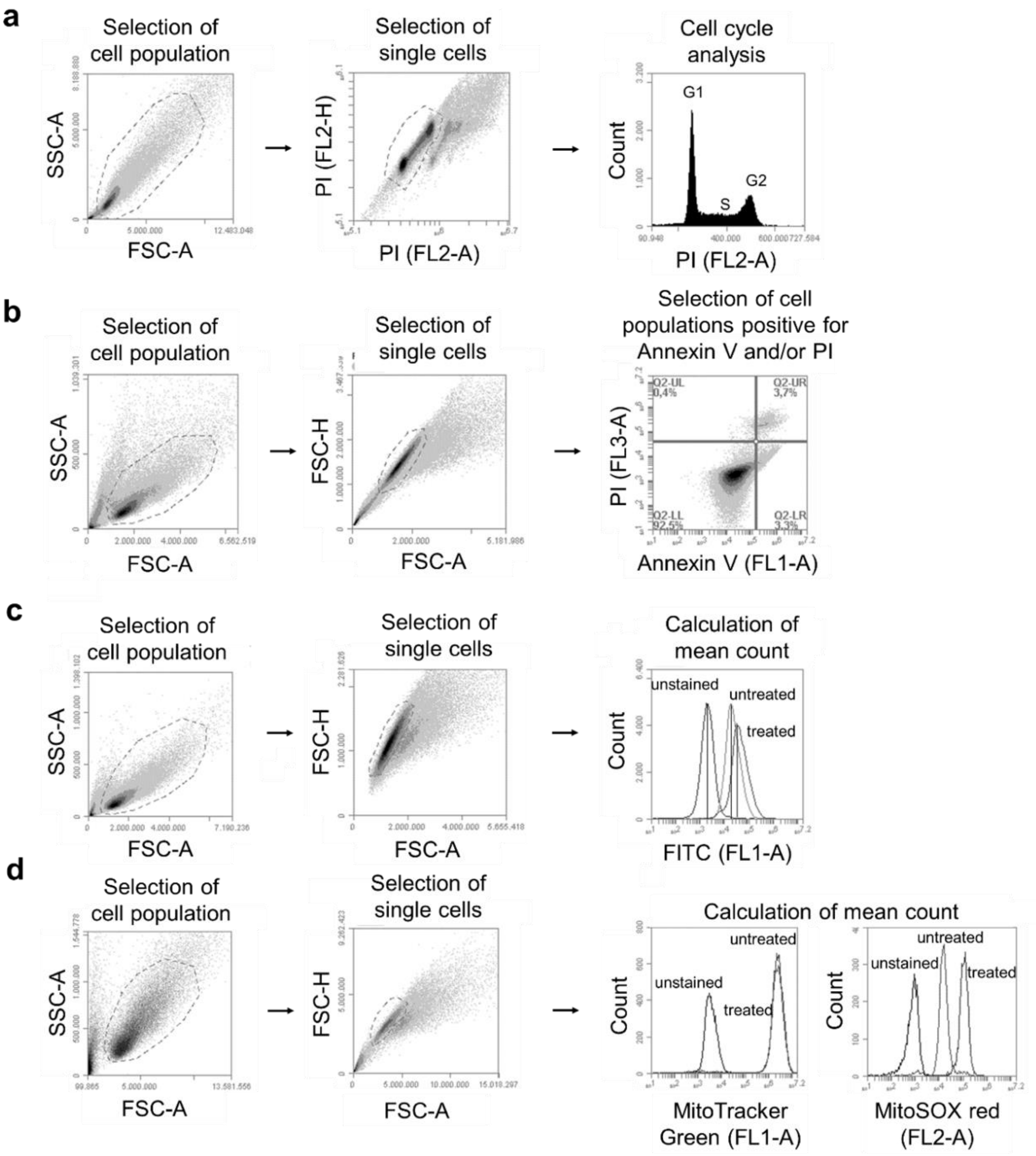

Supplementary Fig. 7 | Examples of flow-cytometric gating strategies
a) Gating strategy for flow-cytometric cell-cycle analysis of EwS cells using PI. b) Gating strategy for Annexin V/PI staining of EwS cells. c) Gating strategy for flow-cytometric measurement of DCF-DA fluorescence in EwS cells. d) Gating strategy for flow-cytometric measurement of MitoSOX Red and MitoTracker Green fluorescence in EwS cells.

Supplementary Table 1 | Characteristics of the intronic *SOX6*-associated GGAA-mSat
Summary of the number of consecutive GGAA-repeats and corresponding relative enhancer activity of the *SOX6*-associated GGAA-mSat in eight cell lines with different *SOX6* expression levels (TC-32 was set as reference).

		GGAA-repeats			Enhancer activity			
**Cell line**	**Average *SOX6* expression (% of TC-32)**	**Allele A**	**Allele B**	**Average number of GGAA-repeats**	**Allele A**	**Allele B**	**Average enhancer activity**	**Average enhancer activity (rel. to TC-32)**
**TC-32**	100	11	13	12	82	175	129	100
**RDES**	77	10	11	10.5	73	127	100	78
**POE**	62	10	14	12	48	93	70	54
**SK-N-MC**	30	8	11	9.5	36	76	56	44
**ORS**	32	10	10	10	50	45	48	37
**EW17**	26	9	10	9.5	25	50	38	29
**EW7**	7	10	11	10.5	76	86	81	63
**A673**	7	8	11	9.5	32	41	37	28

Supplementary Table 2 | Oligonucleotide sequences

**siRNAs**	**Sense (5′→ 3′)**	**Antisense (5′→ 3′)**
Sigma-Aldrich MISSION® siRNA Universal Negative Control #1 (siCtrl)	No sequence given	No sequence given
siTXNIP	CAUCCUUCGAGUUGAAUAUdTdT	AUAUUCAACUCGAAGGAUGdTdT
**shRNAs**	**Top (5′→ 3′)**	**Bottom (5′→ 3′)**
shCtrl	CCGGCAACAAGATGAAGAGCACCAACTCGAGTTGGTGCTCTTCATCTTGTTGTTTTT	AATTAAAAACAACAAGATGAAGAGCACCAACTCGAGTTGGTGCTCTTCATCTTGTTG
shSOX6_1	CCGGCCAGCCCTGTAACTCAAGTTACTCGAGTAACTTGAGTTACAGGGCTGGTTTTTG	AATTCAAAAACCAGCCCTGTAACTCAAGTTACTCGAGTAACTTGAGTTACAGGGCTGG
shSOX6_2	CCGGCCAGTGAACTTCTTGGAGAAACTCGAGTTTCTCCAAGAAGTTCACTGGTTTTTG	AATTCAAAAACCAGTGAACTTCTTGGAGAAACTCGAGTTTCTCCAAGAAGTTCACTGG
shSOX6_3	CCGGTGGTCTTAATTGTTTCGTAAACTCGAGTTTACGAAACAATTAAGACCATTTTTG	AATTCAAAAATGGTCTTAATTGTTTCGTAAACTCGAGTTTACGAAACAATTAAGACCA
shEF1 (EWSR1-FLI1)	CCGGGCAGCAGAACCCTTCTTATGACTCGAGTCATAAGAAGGGTTCTGCTGCTTTTTG	AATTCAAAAAGCAGCAGAACCCTTCTTATGACTCGAGTCATAAGAAGGGTTCTGCTGC
**PCR primer**	**Forward (5′→ 3′)**	**Reverse (5′→ 3′)**
RPLP0	GAAACTCTGCATTCTCGCTCC	GGTGTAATCCGTCTCCACAG
SOX6	TTCCCCGACATGCATAACTC	AAGTGGATCTTGCTTAGCCG
EWSR1-FLI1	GCCAAGCTCCAAGTCAATATAGC	GAGGCCAGAATTCATGTTATTGC
E2F8	ACAGAATGGAGAACGAAAAGGA	TTGGTAGGTGTGGTTAAAGGG
DEPDC1	GGCCAATACAAGTAAACGTGG	CATCTCGTTCAAATCCAACATAAGT
CDCA3	ACTGGAGGGTCTTAAACATGC	ACTTCACTCAGCTGTTTCACC
TXNIP	GATCTGAACATCCCTGATACCC	CATCCATGTCATCTAGCAGAGG
SOX6-GGAA-mSat	CTAGCCCGGGCTCGAGGAGATGTGTCAGCAGTCAATCCA	GATCGCAGATCTCGAGGGCAGTCCAGGATGTTCTGAATAA
cDNA of *TXNIP*	ATTAGCTAGCGCCACCATGGTGATGTTCAAGAAGATCAAGTC	GCGGCGTTAATTAATCACTGCACATTGTTGTTGAGG
eGFP	ATTAGAATTCATGGTGAGCAAGGGCGAG	ATTAGCGGCCGCTTACTTGTACAGCTCGTCCATGC
cDNA of *SOX6*	ACGTATGTCGAGGTAGGCGT	TTCGTCTGACGTGGCAGC
**Sequencing primer**	**Forward (5′→ 3′)**	**Reverse (5′→ 3′)**
SOX6-GGAA-mSat	CTTTATGTTTTTGGCGTCTTCCA	
pLKO-TET-ON	GGCAGGGATATTCACCATTATCGTTTCAGA	
TXNIP-Cumate-System	ATGGTGATGTTCAAGAAGATCAAGTC	AAAGCCTTCACCCAGTAGTC
Modified pTRIPZ vector	ACGTATGTCGAGGTAGGCGT	TTCGTCTGACGTGGCAGC
