# Supplementary material for: Oncogenic hijacking of a developmental transcription factor evokes vulnerability toward oxidative stress in Ewing sarcoma
Source: Nat Commun. 2020 May 15;11:2423. doi: 10.1038/s41467-020-16244-2 (PMC7228971; doi:10.1038/s41467-020-16244-2)
Supplement: Supplementary file 3 — Description of Additional Supplementary Information [file 41467_2020_16244_MOESM3_ESM.pdf]

Description of Additional Supplementary Files

File Name: Supplementary Data 1
Description: Processed data from Affymetrix Clariom D microarrays analyzing splicing events.

File Name: Supplementary Data 2
Description: Most significant differentially expressed genes (absolute FC>1; gene expression >7) comparing shRNA-mediated knockdown of SOX6 to treatment with a non-targeting shControl in two EwS cell lines.

File Name: Supplementary Data 3
Description: Top 100 results of gene-set enrichment analysis of downregulated genes upon shRNA-mediated knockdown of SOX6 in two different EwS cell lines as compared to treatment with a non-targeting shControl.
