# Supplementary material for: Oncogenic hijacking of a developmental transcription factor evokes vulnerability toward oxidative stress in Ewing sarcoma
Source: Nat Commun. 2020 May 15;11:2423. doi: 10.1038/s41467-020-16244-2 (PMC7228971; doi:10.1038/s41467-020-16244-2)
Supplement: Supplementary file 7 — Reporting summary [file 41467_2020_16244_MOESM7_ESM.pdf]

nature research

Corresponding author(s): Thomas G. P. Grünewald

Last updated by author(s): Apr 9, 2020

Reporting Summary

Nature Research wishes to improve the reproducibility of the work that we publish. This form provides structure for consistency and transparency in reporting. For further information on Nature Research policies, see Authors & Referees and the Editorial Policy Checklist .

Statistics

For all statistical analyses, confirm that the following items are present in the figure legend, table legend, main text, or Methods section.

n/a	Confirmed	
☐	☒	The exact sample size (*n*) for each experimental group/condition, given as a discrete number and unit of measurement
☐	☒	A statement on whether measurements were taken from distinct samples or whether the same sample was measured repeatedly
☐	☒	The statistical test(s) used AND whether they are one- or two-sided<br>*Only common tests should be described solely by name; describe more complex techniques in the Methods section.*
☐	☒	A description of all covariates tested
☐	☒	A description of any assumptions or corrections, such as tests of normality and adjustment for multiple comparisons
☐	☒	A full description of the statistical parameters including central tendency (e.g. means) or other basic estimates (e.g. regression coefficient) AND variation (e.g. standard deviation) or associated estimates of uncertainty (e.g. confidence intervals)
☐	☒	For null hypothesis testing, the test statistic (e.g. *F*, *t*, *r*) with confidence intervals, effect sizes, degrees of freedom and *P* value noted<br>*Give P values as exact values whenever suitable.*
☒	☐	For Bayesian analysis, information on the choice of priors and Markov chain Monte Carlo settings
☒	☐	For hierarchical and complex designs, identification of the appropriate level for tests and full reporting of outcomes
☐	☒	Estimates of effect sizes (e.g. Cohen's *d*, Pearson's *r*), indicating how they were calculated

Our web collection on statistics for biologists contains articles on many of the points above.

Software and code

Policy information about availability of computer code

Data collection	GraphPad PRISM 5, Microsoft Excel v16.16.5, CFX Manager v3.1, BD Accuri C6 Cytometer (v.227)
Data analysis	GraphPad PRISM 5, Microsoft Excel v16.16.5, CFX Manager v3.1, MSigDBv5.2, BD Accuri C6 Cytometer (v.227), Transcriptome Analysis Console v4.0, Clariom D Array version 2

For manuscripts utilizing custom algorithms or software that are central to the research but not yet described in published literature, software must be made available to editors/reviewers. We strongly encourage code deposition in a community repository (e.g. GitHub). See the Nature Research guidelines for submitting code & software for further information.

Data

Policy information about availability of data

All manuscripts must include a data availability statement. This statement should provide the following information, where applicable:

- Accession codes, unique identifiers, or web links for publicly available datasets
- A list of figures that have associated raw data
- A description of any restrictions on data availability

The microarray data were deposited at the National Center for Biotechnology Information (NCBI) GEO database under the accession code GSE120576. The microarray, DNase-Seq and ChIP-Seq data referenced during the study are available in a public repository from the GEO website (https://www.ncbi.nlm.nih.gov/geo/); accession codes: GSE34620, GSE37371, GSE88826, GSE64686, GSM736570, and GSE61944. The source data underlying Figures 1a–b, 2a–c, 2e–f, 3a–d, 3f–i, 4a–k, 5a–g, 6a–h and Supplementary Figures 1a, 2a–c, 3a–j, 4a–c, 5a–c and 6a–c are provided as a Source Data file. All the other data supporting the findings of this study are available within the article and its supplementary information files and from the corresponding author upon reasonable request.

Field-specific reporting

Please select the one below that is the best fit for your research. If you are not sure, read the appropriate sections before making your selection.

- [x] Life sciences
- [ ] Behavioural & social sciences
- [ ] Ecological, evolutionary & environmental sciences

For a reference copy of the document with all sections, see nature.com/documents/nr-reporting-summary-flat.pdf

Life sciences study design

All studies must disclose on these points even when the disclosure is negative.

Sample size	In vitro sample sizes were based on previous similar studies that have given statistical results. For in vivo experiments, sample sizes were predetermined using power calculations with beta=0.8 and alpha<0.05 based on preliminary data and in compliance with the 3R system (replacement, reduction, refinement).
Data exclusions	No data were excluded from the analyses.
Replication	All attempts at replication yielded similar results. Precise information on the number of biologically independent replicates of each experiment are given in the corresponding figure legends.
Randomization	For in vivo experiments, mice were numbered and randomized into control and treatment groups with similar distributions of male and female animals. All mice had a similar age at start of the given experiment (10-12 weeks). For in vitro experiments, randomization and controlling of covariates was not necessary or advised, since the highly standardized cell culture conditions (see Methods section) largely eliminate biological noise/variation across experiments.
Blinding	Histopathological analysis of human tissue samples was carried out in a blinded fashion. For animal treatment studies, drug adminsitration was only possible in an unblinded manner due to ethical regulations and monitoring of potential adverse effects. For histopathological assessment of the xenografts, the investigators were blinded with respect to group allocation during data collection and analysis.

Reporting for specific materials, systems and methods

We require information from authors about some types of materials, experimental systems and methods used in many studies. Here, indicate whether each material, system or method listed is relevant to your study. If you are not sure if a list item applies to your research, read the appropriate section before selecting a response.

Materials & experimental systems

n/a	Involved in the study	
[ ]	[x]	Antibodies
[ ]	[x]	Eukaryotic cell lines
[x]	[ ]	Palaeontology
[ ]	[x]	Animals and other organisms
[ ]	[x]	Human research participants
[x]	[ ]	Clinical data

Methods

n/a	Involved in the study	
[x]	[ ]	ChIP-seq
[ ]	[x]	Flow cytometry
[x]	[ ]	MRI-based neuroimaging

Antibodies

Antibodies used	For immunohistochemistry analyses, the following antibodies were used:<br>Anti-SOX6 (HPA003908, Atlas Antibodies), anti-cleaved caspase 3 (#9661, Cell Signaling), anti-KI67 (#275R-15, Cell Marque), anti-TXNIP (EPR14774, Abcam), anti-rabbit IgG (MP-7401, Vector Laboratories).<br><br>For Western Blot analyses, the following antibodies were used:<br>Mouse monoclonal anti-SOX6 (SC-393314; Santa Cruz), rabbit monoclonal anti-TXNIP (ab188865, Abcam), mouse anti-GAPDH (Sc-32233, Santa Cruz), anti-rabbit IgG (R1364HRP, OriGene), anti-mouse IgG (H+L) (W402b, Promega).
Validation	Specificity of the used antibodies against human antigens was tested by knockdown experiments of the corresponding antigen and subsequent Western blot and/or immunohistochemistry analyses. In addition, antibodies were validated for the given application by the manufacturer as stated in the respective datasheets (see links) and references (see DOIs):<br><br>* anti-SOX6 (HPA003908, Atlas Antibodies): developed and validated by the Human Protein Atlas (HPA) project (www.proteinatlas.org), Ref: DOI:10.1093/nar/gks466<br><br>* anti-cleaved caspase 3 (#9661, Cell Signaling), https://www.cellsignal.de/products/primary-antibodies/cleaved-caspase-3-asp175-antibody/9661, Ref: DOI:10.3892/ijmm.2020.4453

* anti-KI67 (#275R-15, Cell Marque), https://www.cellmarque.com/antibodies/CM/108/Ki-67_SP6, Ref: DOI:10.1097/00005072-199810000-00005

* anti-TXNIP (EPR14774, Abcam), https://www.abcam.com/txnip-antibody-epr14774-bsa-and-azide-free-ab215366.html, Ref: DOI:10.1016/j.redox.2018.02.013

* anti-rabbit (MP-7401, Vector Laboratories), https://vectorlabs.com/immpress-hrp-horse-anti-rabbit-igg-peroxidase-polymer-detection-kit.html, Ref: DOI:10.1111/bph.1484

* anti-SOX6 (SC-393314; Santa Cruz), https://datasheets.scbt.com/sc-393314.pdf, Ref: DOI:10.3324/haematol.2018.206227

* anti-TXNIP (ab188865, Abcam), https://www.abcam.com/txnip-antibody-epr14774-ab188865.html, Ref: DOI:10.1186/s13041-019-0463-2

* anti-GAPDH (Sc-32233, Santa Cruz), https://datasheets.scbt.com/sc-32233.pdf, Ref: DOI:10.1016/j.yjmcc.2019.01.029

* anti-rabbit IgG (R1364HRP, OriGene), https://cdn.origene.com/datasheet/r1364hrp.pdf, Ref: DOI:10.2164/jandrol.109.008623

* anti-mouse IgG (H+L) (W402b, Promega), https://www.promega.de/products/protein-detection/primary-and-secondary-antibodies/anti_mouse-igg-h-and-l-hrp-conjugate/?catNum=W4021#specifications , Ref: DOI:10.1074/jbc.M112.343566

Eukaryotic cell lines

Policy information about cell lines

Cell line source(s)

The neuroblastoma cell line SK-N-AS as well as HEK293T were purchased from ATCC. Human Ewing sarcoma cell lines and other cell lines were provided by the following repositories and/or sources: A673 cells were purchased from ATCC. MHH-ES1, RDES, RH-1, SK-ES1 and SK-N-MC and cells were provided by the German collection of Microorganism and Cell Cultures (DSMZ). CHLA-10, CHLA-25, CHLA-32, CHLA-57, CHLA-99, COG-E-352, TC-32, TC-71 and TC-106 were kindly provided by the Children's Oncology Group (COG), and ES7, EW1, EW3, EW7, EW16, EW17, EW18, EW22, EW24, MIC, ORS, POE, SK-PN-DW, SK-PN-LI and STA-ET1 as well as rhabdomyosarcoma (Rh4, Rh36) and neuroblastoma (TGW) cell lines cells were provided by O. Delattre (Institute Curie, Paris). A673/TR/shEF1 cells were provided by J. Alonso (Madrid, Spain). Human osteosarcoma cell lines SAOS-2 and U2OS were provided by DMSZ, and the MSC cell line MSC-52 was generated from bone marrow of a Ewing sarcoma patient (provided by U. Dirksen; Essen, Germany).

Authentication

Cell lines were authenticated by STR-profiling and if applicable by detection of specific fusion oncogenes by qRT-PCR.

Mycoplasma contamination

All cell lines were tested negative for mycoplasma contamination by nested PCR.

Commonly misidentified lines
(See ICLAC register)

SK-N-MC: The SK-N-MC cell line is listed in the database of commonly misidentified cell lines, ICLAC (http://iclac.org/databases/cross-contaminations), as it was initially described to be a neuroblastoma cell line. Indeed, it is a Ewing sarcoma cell line expressing the pathognomonic fusion oncogene EWSR1-FLI1.

Animals and other organisms

Policy information about studies involving animals; ARRIVE guidelines recommended for reporting animal research

Laboratory animals

NOD.Cg-Prkdcscid Il2rgtm1Wjl/SzJ (NSG) mice were obtained from Jackson Laboratory via Charles River Laboratories and bred under a MTA with LMU Munich. All NSG mice (male and female) had an age between 10 and 12 weeks at start of the experiments. Mice were housed in individually ventialed cages (IVC) under specific pathogen-free (SPF) conditions with strict dark/light cycles (darkness from 6 PM to 6 AM), an ambient temperature of 21-23°C and a humidity of 45-65%.

Wild animals

No wild animals were used in this study.

Field-collected samples

No field-collected samples were used in the study.

Ethics oversight

Experiments were approved by the government of Upper Bavaria and conducted in accordance with ARRIVE guidelines, recommendations of the European Community (86/609/EEC), and UKCCCR (guidelines for the welfare and use of animals in cancer research).

Note that full information on the approval of the study protocol must also be provided in the manuscript.

Human research participants

Policy information about studies involving human research participants

Population characteristics

Human tissue samples for each tumor entity as well as normal tissue types were retrieved from the archives of the of the Institute of Pathology of the LMU Munich (Germany). This study included samples from 10 cancer entities and 18 normal tissue types (for details see Fig. 1b). Except for the pathological diagnosis and the information that samples were from pre-treatment

	conditions, no clinical information such as age at diagnosis and sex were collected, since these data were not relevant for the comparative expression analysis of SOX6 across cancer entities and normal tissue types. For Ewing sarcoma patients who donated parts of their tumors for generation of PDX models, clincial information was available. Overall, the patients' clincial features and demographics were typical of Ewing sarcoma. These patients had an average age of 9.6 years (range: 4–17 years) at diagnosis. The ratio of males/females was 1.
Recruitment	Human tissue samples were retrieved from the archives of the Institute of Pathology of the LMU Munich (Germany) comprising the years 2008-2018. All available samples for the given tumor entity and representative samples of normal tissue types were collected and screend by a pathologist for sufficient material on histological sildes. Since the observed expression pattern of SOX6 at the protein level in these samples (Fig. 1b) was highly similar to the SOX6 mRNA expression pattern observed in well-curated and publcily available gene expression microarray data (Fig. 1a) (Baldauf MC et al. 2018 OncoImmunology), selection bias appears unlikely. All Ewing sarcoma patients who donated parts of their tumors for PDX model generation were recruited between 2013 and 2018. PDX models were established whenever enough viable tumor material was available after resecton and processing by pathologists. The sex of the recipient mice was chosen accordingly to the donor patient.
Ethics oversight	Human tissue samples were analyzed at the Institute of Pathology of the LMU Munich (Germany) with approval of the institutional review board. All patients/guardians provided written informed consent. All analyses were approved by the ethics committee of the LMU Munich (approval no. 18-481 UE).

Note that full information on the approval of the study protocol must also be provided in the manuscript.

Flow Cytometry

Plots

Confirm that:

- [x] The axis labels state the marker and fluorochrome used (e.g. CD4-FITC).
- [x] The axis scales are clearly visible. Include numbers along axes only for bottom left plot of group (a 'group' is an analysis of identical markers).
- [x] All plots are contour plots with outliers or pseudocolor plots.
- [x] A numerical value for number of cells or percentage (with statistics) is provided.

Methodology

Sample preparation	Human Ewing sarcoma cells were trypsinized from cell cluture dishes, washed with PBS, and stained for AnnexinV-FITC and propidium iodine (PI) using the BD Pharmingen Apoptosis detection kit I acccording to the manufacturer's protocol or incubated with DCF-DA or with MitoSox Red/MitoTracker Green according to the manufacturer's protocol.
Instrument	Accuri C6 flow cytometer.
Software	Accuri C6 CFlow Plus.
Cell population abundance	All flow cytometry experiments were done in pure human Ewing sarcoma cell lines without admixture of any other cell type.
Gating strategy	Gating included forward and side scatter plots for gating living cells (i.e. exclusion of debris), and subsequent filtering for singletons. An example for the gating strategy is given in Supplementary Figure 7.

- [x] Tick this box to confirm that a figure exemplifying the gating strategy is provided in the Supplementary Information.
